# The History and Capabilities of Herbal Simples

**Published:** 1891-06-27

**Authors:** W. T. Fernie


### The History and Capabilities of Herbal Simples.

By W. T. Fernie, M.D.

### XXXIII.—THE ELDER—ELECAMPANE.

The Elder *(continued from page 88).*

From the Rev. W. Symonds we learn that an open space now seen in Malvern Chase was formerly called Eldersfield, from the abundance of elder trees which grew there. "The flowers were noted for eye ointments, and the berries for honey-rob and black pigments." "Mary of Eldersfield, the daughter of Bolingbroke, was famous for her knowledge of herb pharmacy, and for the efficacy of her nostrums." Again, Abraham Munting, of Amsterdam, who wrote a whole book, in 1695, about the Great Water Dock, averred that a simple decoction of that root will not cure scurvy. He, therefore, gave the following receipt, which "the world never had before, and which is more precious than gold, so that the medicine should be kept as an inestimable treasure against all suspicions of the scurvy: Take of saffron, black pepper, and gentian root of each three ounces, of the dock root six ounces, and of strong elder vinegar two quarts. Mix, and infuse all these in an earthern glazed vessel, at milk heat, in hot ashes for two hours; then set it by for use." "Give as a dose from three to six ounces every morning for fourteen, or twenty, or thirty days together." "It is not only a good wine in the scurvy but likewise in all nervous disorders."

John Evelyn in his "Silva" (1729) said of the elder: "If the medicinal properties of its leaves, bark, and berries were fully known I cannot tell what our countrymen could ail for which he might not fetch a remedy from every hedge, either for sickness or for wounds." "The buds boiled in water-gruel have effected wonders in a fever, and an extract composed of the berries greatly assists longevity." "Indeed (so famous is the story of Neander), this is a catholicum against all infirmities whatever." "The leaves are somewhat rank of smell, and, therefore, not commendable in sallet; they are otherwise—as, indeed, is the entire shrub—of a very sovereign virtue. The spring buds are excellently wholesome in pottage; and small ale in which elder flowers have been infused are esteemed by many so salubrious that this is to be had in most of the eating-houses about our town." Shakespeare tells in "Henry V." about "a perilous shot out of an elder gun"; and, again, in the "Merry Wives of Windsor," speaks of the braggart, Dr. Caius, as having a "heart of elder," alluding to the light, untrustworthy pith of the tree, as compared with a "heart of oak, solid to the core."

An early English herbal directs to "put three drops of blood from a wart into an eldern leaf, and burie it in the earth, when the warts will vanish away." Lord Bacon also said "the like is done by rubbing of warts with a green elder stick, and then burying the stick to rot in mud." Various superstitions have attached themselves in England to the elder bush. The Tree-Mother has been supposed to inhabit it; and on this account until recently many persons have refused to cut it down. A tale runs that a certain elf which had tenanted an elder tree from the wood of which the cradle was made in which an infant was placed, continued to pull the child by the legs, and to tease it until it was taken out of the cot. Thorpe mentions an elder bush in a village court which used to move about at dusk, and to peep in at windows. And it has been long believed that refuge may be safely taken under an elder tree in a thunder-storm, because the cross was made of that wood, and so the lightning never strikes it. In earlier times elder was buried with a corpse to protect it from witches, and even now at a funeral the driver of the hearse commonly has his whip handle made of elder wood. It is curious that the caterpillar of the death's-head moth feeds chiefly on elder leaves. The great Boerhaave always took off his hat when passing an elder bush. Douglas Jerrold once at a well-known tavern ordered a bottle of port wine which was to be old, but not elder.

### Elecampane.

"Elecampane," writes William Coles, "is one of the plants whereof England may boast as much as any, for there grows none better in the world than in England, let apothecaries and druggists say what they will." Nowadays it is still cultivated in our gardens as a medicinal and culinary herb, though found wild only in our copses and meadows as of rare and local production. It is a tall, stout, downy plant, from three to five feet high, with broad leaves, and bearing bright yellow flowers of the composite order. The common name, Elecampane, has been derived from *elle*, Danish for fairy, and Campania, the local source of the plant, which is also called elfwort and elfdock. Its botanical appellation is Inula Helenium, the latter of which words commemorates Helen of Troy, from whose tears the herb was supposed to have sprung, or whose hands were full of its leaves when Paris carried her off from Menelaus. The name Inula, or Enula, is a tautological term, being merely an alteration of the Greek *elenium.* Oddly enough the title of this plant has been corrupted to Horse-heal, or Horshele, or Horse heel, through a double blunder, because the word *inula* was misunderstood for *hinnula*, a colt; and the term hellenium was thought to have something to do with heels or healing, and solely on this account the Elecampane has been employed by apothecaries to heal horses of scabs and sore heels. The herb is of ancient repute, and was well described by Dioscorides. An old Latin distich thus celebrates its virtues:—

"Enula campana reddit præcordia sana,"
Elecampane will the spirits sustain.

Prior to the Norman Conquest, and during the middle ages, its root was much employed here as a medicine, and likewise this was candied and eaten as a sweetmeat. Some fifty years ago the candy was commonly sold in London, being composed largely of sugar and coloured with cochineal. A piece was eaten each night and morning for asthmatical complaints, whilst it was customary when travelling by a river to suck a bit of the root against poisonous exhalations and bad air. The candy may yet be had from our confectioners, but now containing no more of the plant than there is of barley in barley sugar. "The flowers of Elecampane," says Gerarde, "are in all their bravery during June and July; the roots should be gathered in the autumn. This plant is good for an old cough, and for such as cannot breathe freely unless they hold their necks upright; also it is of great value when given in a looch, which is a medicine to be licked on. It voids out thick clammy humours which stick in the chest and lungs." Galen says, further, "it is good for passions of the huckle bones, called sciatica." The root is thick and substantial, and has, when sliced, a fragrant aromatic odour. Chemically, it contains a crystalline principle, resembling camphor, and called hellenine; also a starch named inuline, which is peculiar because not soluble in water, alcohol, or ether; together with a volatile oil, a resin, albumen, and acetic acid. It has proved particularly useful in coughs and chronic pectoral complaints, by acting as a sudorific, and promoting expectoration. Also it counteracts the acidity of gouty indigestion, and regulates the monthly illnesses of women. The French use it in the distillation of absinthe, and call it "l'aulnee"—d'un lieu planté d'aulnes, ou elle se plait. To make a decoction, half an ounce of the root should be gently boiled for ten minutes in a pint of water and then allowed to cool. From one to two ounces of this may be taken three times in the day. Of the powdered root from half to one teaspoonful may be given for a dose. A medicinal tincture is prepared from the root by certain chemists, and of this thirty or forty drops may be taken at a time with a tablespoonful of cold water. Too large a dose will induce vomiting. Elecampane is specifically curative of a sharp pain affecting the right elbow-joint, and recurring daily; also of a congestive headache through pressure in the lowest bowel. Moreover, just at the present time, when there is so much talk about Koch and his treatment of consumption, it is highly interesting to know that the hellenine of Elecampane is said to be peculiarly destructive to the bacillus of tubercular disease. In classic times the poet Horace related how Fundanius first taught the making of a delicate sauce for dinner by boiling in it the bitter elecampane; and how the Roman sto mach, when surfeited by excess of rich fare, pined for turnips, and the pungent "enulas acidas" from frugal Campania.

